# Revisiting the IGF-1R as a breast cancer target

**DOI:** 10.1038/s41698-017-0017-y

**Published:** 2017-05-01

**Authors:** Roudy Chiminch Ekyalongo, Douglas Yee

**Affiliations:** Masonic Cancer Center, University of Minnesota, MMC 806, 420 Delaware Street SE, Minneapolis, MN 55455 USA

## Abstract

The type I insulin-like growth factor-1 receptor is a well-described target in breast cancer and multiple clinical trials examining insulin-like growth factor-1 receptor have been completed. Unfortunately, monoclonal antibodies and tyrosine kinase inhibitors targeting insulin-like growth factor-1 receptor failed in phase III breast clinical trials for several reasons. First, insulin-like growth factor-1 receptor antibody therapy resulted in hyperglycemia and metabolic syndrome most likely due to disruption of insulin-like growth factor-1 homeostasis and subsequent growth hormone elevation. Growth hormone elevation induces insulin resistance, hence a subsequent elevation of insulin and the potential for activation of insulin receptor. Second, the insulin-like growth factor-1 receptor and insulin receptor are highly homologous in amino acid sequence, structure, and function. These two receptors bind insulin, insulin-like growth factor-1 and insulin-like growth factor-2, to regulate glucose uptake and other cellular functions. Hybrid receptors composed of one chain of insulin-like growth factor-1 receptor and insulin receptor also participate in signaling. Third, since all the monoclonal antibodies were specific for insulin-like growth factor-1 receptor, any pathophysiologic role for insulin receptor was not inhibited. While the insulin-like growth factor-1 receptor tyrosine kinase inhibitors effectively inhibited both insulin-like growth factor-1 receptor and insulin receptor, these drugs are not being further developed likely due to their metabolic toxicities. Insulin-like growth factor-1/2 neutralizing antibodies are still being studied in early phase clinical trials. Perhaps a more comprehensive strategy of targeting the insulin-like growth factor-1 receptor network would be successful. For example, targeting receptor, ligand and downstream signaling molecules such as phosphatidylinositol 3′-kinase or particularly the insulin receptor substrate adapter proteins might result in a complete blockade of insulin-like growth factor-1 receptor/insulin receptor biological functions.

## Introduction

At least 50% of breast tumors have an activated type 1 insulin-like growth factor-1 receptor (IGF-1R).^1^ Several preclinical investigations have associated the activation of IGF-1R by its two natural ligands, insulin-like growth factor-1 (IGF-1) and IGF-2,^2^ as primary risk factors in various types of human diseases^3^ including cancer.^4^ A case for targeting IGF-1R was based on several observations. First, IGF signaling enhanced normal and tumor cell growth, survival, and motility. Second, the IGF-binding proteins (IGFBPs) are widely expressed in breast cancer and linked to outcome.^5^ The IGFBPs regulate^6^ interactions between ligand and receptor and also serve to transport IGF-1 and IGF-2 in extracellular fluids.^7^ Third, sources of IGF-1 and IGF-2 are abundant and available to tumor cells by endocrine sources as well as through autocrine/paracrine production from tumor tissue.^8, 9^

In addition to IGF-1R, insulin receptor (IR) also functions in the IGF-signaling system, especially the fetal A isoform (discussed below). The functional similarity between receptors is high with a high level of conservation between the two receptors.^6^ IGF-1R and IR are approximately 60% identical in amino acid sequence and even higher in the kinase domains. The clearest evidence to illustrate the similar physiologic functions has been shown in tumor-associated hypoglycemia induced by the pathophysiologic elevation of insulin^10, 11^ or IGF-2 from islet^12^ or non-islet tumor cells.^13^ Further evidence of the shared functionality of the systems was the early clinical experience in using IGF-1 as a therapy for type 2 diabetes.^14^

In breast cancer, although both IGFs and insulin have been reported to regulate cell growth, most of the therapeutic agents have targeted IGF-1R function. While stimulation of IGF-1R by IGFs triggers autophosphorylation and subsequent phosphorylation of either insulin receptor substrate-1 (IRS-1) or insulin receptor substrate-2 (IRS-2), it is clear that these adapter proteins are activated by both IR^15^ and IGF-1R.^16^ IRS-1/2 proteins serve as scaffolds to activate other intermediate signaling proteins such as PI3K/AKT/mTOR^17^ and Ras/Raf/MAPK^18^ and this function has been well-reported in all breast cancer subtypes.^19^ The IRS-1/2 activation was reported in estrogen receptor (ER) positive,^20^ human epidermal growth factor receptor 2 (HER2 or c-erbB2) positive,^21^ and triple-negative breast cancer (TNBC).^22^ Thus, the IGF system is linked to all the intrinsic subtypes of breast cancer.^23, 24^

Previous work in our laboratory has demonstrated the expression of IGF-1R adapter proteins, specifically IRS-1, is correlated with poor prognosis in ER positive breast cancer patients.^25^ Both the Endogenous Hormones and Breast Cancer Collaborative Group^26^ and European Prospective Investigation into Cancer and Nutrition cohort^27^ have emphasized the cross talk between ER and IGF-1 receptors increases breast cancer risk. These findings have motivated substantial preclinical and clinical effort into developing drugs to disrupt this signaling system.

Several strategies have been tested to overcome IGF-1R signaling,^28^ including IGF-1R blockade by monoclonal antibodies (mAb), small molecule tyrosine kinase inhibitors (TKIs) of IGF-1R and IR, and ligand neutralizing strategies. For example, we demonstrated the benefits of ligand neutralization by IGFBP-1 in model systems.^29^ More recently ligand neutralizing mAb such as MEDI-573^30^ have been show to decrease the tumorigenic behavior of IGF-1R positive breast cancer. While the neutralizing antibody MEDI-573 has been reported to avoid disruption of the growth hormone (GH)/IGF-1 feedback system, another neutralizing antibody (BI 83645-xentuzumab) induced GH increases in mice.^31^ These ligand neutralization trials are just beginning^32^ as discussed below. In contrast, the inhibition of IGF-1R either by mAbs or TKIs has been well studied in clinical trials.

The mAb and TKIs have not shown benefit when added to conventional endocrine therapy in breast cancer. Several reasons may account for this failure. It is known the blockade of IGF-1R enhances GH levels to induce hyperglycemia,^33^ but hyperinsulinemia is also seen.^34^ Studies have suggested that individual tumors may rely upon IR signaling for growth and proliferation.^35, 36^ In tumors where IR has a pathophysiologic role, anti-IGF-1R mAbs would not be expected to confer a clinical benefit if elevated insulin levels and IR activation are a result of the anti-IGF-1R therapy. To address this, dual treatment with IGF-1R/IR inhibitors may be the appropriate approach to avoid compensatory cross talk between IGF-1R and IR.^37^ The IGF-1R TKIs showed inhibition of both IGF-1R and IR^38, 39^ and even had activity in a hyperinsulinemic mouse model.^40^ However, these drugs are not being developed due to concerns about affecting host glucose uptake.

The motivation to develop an alternative molecular strategy, beyond just targeting ER-α, in ER positive breast cancer was supported by several preclinical observations. Estradiol (E2) and its receptor ER enhance the expression and activation of the IGF-1R^41, 42^ tumorigenic signaling cascade including upregulation of IRS-1 resulting in enhanced phosphatidylinositol 3′-kinase (PI3K) pathways.^43^ Therefore, it was believed that treating breast cancer with dual targeting of ER and IGF-1R could improve clinical benefit compared with targeting ER alone. Unfortunately, this strategy failed to show clinical benefit in the overall breast cancer patient population^44^ as demonstrated by no improvement in disease-free survival when the IGF-IR antibody ganitumab was added to endocrine treatment.^33^ Considering the lack of benefit in this phase III trials, several drug sponsors terminated their IGF-1R drug development programs^37^ in recent years. Although the response rate of anti-IGF-1R in clinical trials was disappointing, there are several strong pieces of evidence in preclinical models that defined the ability of anti-IGF-1R mAb to block the growth and migration of breast cancer cells as therapeutic potencies.^9, 45^ Also, they appeared to be a benefit in patients who did not have evidence of pre-existing glucose intolerance as measured by glycosylated hemoglobin.^46^ Indeed, all the studies as described have motivated investigators to search for an alternative approach to maximize the therapeutic effect of anti-IGF-1R treatment.

## Molecular elements of cross talk between IR and IGF-1R

There is a growing body of evidence describing the physiological and therapeutic relevance of the functional similarities between IR and IGF-1R in many diseases including diabetes and cancer.^47^ While the genetic features and mRNA sequence of both receptors are distinguishable, for instance, the IGF-1R gene located on chromosome 15q26.3 is encoded by 25-exons, while InsR gene is found on chromosome 19p13.3-p13.2 and encoded by 22-exons. Yet, the homology of amino acid sequences of IR and IGF-1R is high. The ligand-binding domains of both receptors, namely, the C-terminus of the α-chain on the cell surface, are estimated to be 55% related. While their tyrosine kinase domain (β-chain) in the internal leaflet of plasma membrane has approximately 72% similarity,^48^ the ATP-binding domains in both receptors are 100% identical.^49^

**Fig. 1:**
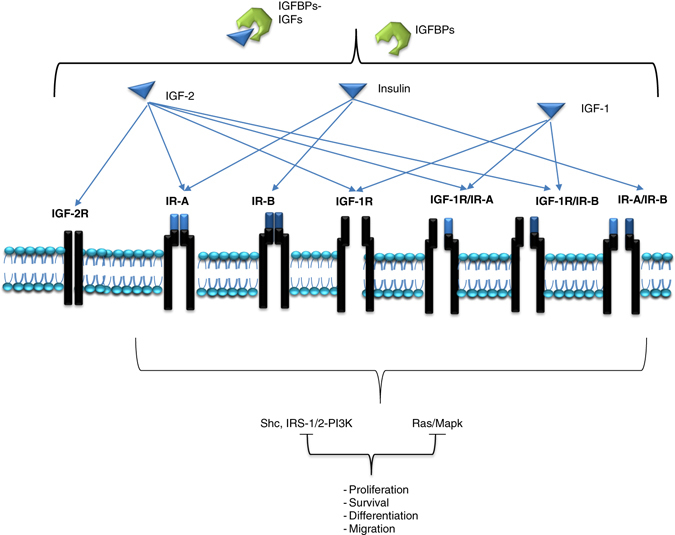
Schematic representation of the type I IGF-1R and IR signaling network. The network is composed of two principal receptors and three ligands. The receptors are transcribed from a single gene, IGF-1R, and IR. In addition, InsR has two isoforms generated by splice variants of the IR gene, IR-A and IR-B. Each gene transcribes a single protein which is then processed into an α and β subunit. These subunits may form a holoreceptor (IGF-1R, IR-A, IR-B) or the units can form heterodimeric hybrid receptors (IGF-1R/IR-A, IGF-1R/IR-B, IR-A/IR-B). The type II IGF receptor (IGF-2R) is not a signaling receptor, but has a high affinity for IGF-2 and is thought to result in the degradation of IGF-2. The IGFs are also complexed with IGFBPs in extracellular spaces. Both IGF-1 and IGF-2 exert their effects through autocrine, paracrine, and endocrine mechanisms, and can activate the IGF-1R and IR pathways. All IGF-1R network receptors are partially similar in their ligand-biding domain, while their intracellular ATP tyrosine kinase-binding domains are nearly identical. The binding of each receptor by their ligands induce the phosphorylation of Shc and IRS-1/2. These adapter proteins transmit signals through the PI3K–AKT1–mTOR pathway or Ras/MAPK pathway to control cell growth, survival, migration, and differentiation

The receptors were first identified as holoreceptors containing αβ chains transcribed from a single gene, but it is also evident hybrid receptors can form from the two separate gene products (Fig. 1). This hybrid receptor (IGF-1/IR) is made from the linkage of the αβ subunits, encoded by both the InsR and IGF-1R genes, into a heterodimeric receptor.^37^ Isoforms of both receptors exist, but the most important isoform to cancer biology is the insulin receptor-alpha (IR-A), which is also the fetal form of IR. The simplest approach to distinguish these receptor families is the analysis of the binding affinity to their common ligands. For instance, IGF-1R binds IGF-1 with high affinity and has lower affinity to IGF-2 and insulin (Fig. 1), but IR-A has a higher affinity for insulin and IGF-2 and not for IGF-I.^50^ Additional data indicate IGF-1R/IR hybrid receptors have higher affinity to IGF-1^51^; thus, this affinity for multiple receptors may allow IGF-1 to be tumorigenic in many types of cancer.

While the higher binding affinity of insulin to IR-A was first characterized as a pathway for glucose regulation during fetal and embryological life, IR-A also is expressed in many types of cancer.^30^ In contrast, the insulin receptor-beta (IR-B) isoform is preferentially expressed in adult tissue to control insulin signaling,^48^ although there is evidence that IR-B also has a role in cancer.^52^ The homology of both ligands, IGFs, and insulin is estimated to be at 50% which partially contributes to the cross-activation of IGF-1R and IR.^53^ Even though both ligand families are involved in similar cell signaling pathways, their function and activity in extracellular compartments appears different. For instance, among IGFBPs (see “Introduction”), IGFBP-3 is the predominant binding partner for IGF-1 in the serum as compared with IGFBPs-1, IGFBPs-2, IGFBPs-4, IGFBPs-5, IGFBPs-6.^54^ This ability is simply due to the serum abundance of IGFBP-3,^55^ thereof the ternary complex of IGF-1, IGFBP-3, and an acid-labile subunit is principally seen to modulate antiproliferative activity in breast cancer.^56^ IGFBP-3 has been tested in preclinical models to inhibit IGF-action.^57^

While the increase of free IGF-2 is associated with the suppression of insulin, IGF-1, and GH serum concentration,^58^ the binding of IGF-2 to IGFBPs prevents excessive free IGF-2 in serum to cause hypoglycemia.^59^ Unlike IGFs, insulin selectively inhibits the transcription of both genes IGFBP-1^60, 61^ and IGFBP-2.^62^ Of note, insulin is known as a primary regulator of glucose uptake, but this hormone also enhances proliferation in breast cancer has been described.^63, 64^ This evidence demonstrates the complex interactions between binding proteins, ligands, and receptors in regulating tumor cell biology.

## Status of clinical trials involving anti-IGFs and IGF-1R mAbs

The preclinical findings that support IGF-1 and its receptor IGF-1R as potential therapeutic targets led to the initiation of many clinical trials in the last decade (Table 1). To estimate the scope of the IGF-targeted therapies, the clinicaltrial.gov database has recorded a total of 625 clinical trials where IGF-1 was cited either as a diagnostic marker or therapeutically targeted molecule in several diseases including cancer. However, few clinical trials used a ligand neutralizing approach. For this purpose, there are only two IGF-1/2 neutralizing mAbs that are under investigation in clinical trials.^37^ One of the neutralizing mAb is MEDI-573; the pharmacodynamics of this anti-IGF-1/2 mAb has been reported in phase I clinical trial in patients with advanced solid tumor. This report showed suppression of IGF-1 and IGF-2 without defining a dose-limiting toxicity including metabolic disorders.^65^

**Table 1:** Current potential anti-IGFs and IGF-1R mAb in breast cancer trials

Drug type						
	Breast cancer indication	IHC-criteria	Phase of trial	Drugs supplements	Estimated date/clinical trial phase	Reference ID # Clinical Trials.gov
*IGF-1 and IGF-2 neutralizing mAbs*						
Dusigitumab^*^ (MEDI-573)	Metastatic	HR+/HER2−	*n* = 188 Phase II	Aromatase inhibitor	06/2011 to 09/2017	NCT01446159
Xentuzumab (BI836845)^*^	Metastatic	HR+/HER2−	*n* = 174 Phase II	MTOR and Aromatase inhibitor	05/2014 to 04/2018	NCT02123823
*IGF-IR mAb*						
Cixutumumab (IMC-A12)^*^	Locally advanced Metastatic	HER2/neu+	*n* = 64 Phase II	Capecitabine	07/2008 to Ongoing	NCT00684983
	Metastatic		*n* = 48 Phase II	MTOR inhibitors	10/2008 to Ongoing	NCT00699491
R1507^ώ^	Metastatic		*n* = 8 Phase II	None	07/2009 to 12/2010	NCT00796107
Dalotuzumab^*^ (MK0646)	Metastatic	HR+/HER2−Ki67 ≥ 15%	*n* = 84 Phase II	Aromatase inhibitors	10/2012 to 03/2017	NCT01605396
Ganitumab (AMG479)^*^	Stage II–III	HR+/HER2+, Mamma Print low	*n* = 1920 Phase II	Anti-hyperglycemic	03/2010 to 05/2018	NCT01042379

*HR+* hormone receptor positive that includes estrogen positive, progesterone receptor positive or both, *HER2+* human epidermal growth factor receptor 2 positive, *HER2+* human epidermal growth factor receptor 2 negative, ^*ώ*^clinical trial accrual was suspended as reported by clinicaltrial.gov, *no clinical trial results published by clinicaltrial.gov as this is an ongoing clinical trial

Due to the work of the previous phase I studies, MEDI-573 is currently being tested in phase II clinical trial for late stage breast cancer (NCT01446159) and is expected to be completed in September 2017. BI836845 is another mAb targeted against IGF-1/2 and studied in preclinical models.^31^ In contrast to MEDI-573, BI836845 administration increased IGF-1 serum concentrations. Detailed analysis showed the increased IGF-1 was found in complex with BI836845 in the presence of lower IGFBP-3 expression. Alternative studies have demonstrated BI836845 prevented ligand activation of IGF-1R/IR-A, then reduced cell proliferation.^66^ This phenomenon observed in preclinical studies along with other positive outcomes during the phase I trial have allowed BI836845 to continue to clinical trial phase II, where it is used in combination with an mTOR inhibitor (everolimus) and an aromatase inhibitor (exemestane) in metastatic breast cancer patients (NCT02123823).

In breast cancer, there are a total of 22 clinical trials reported in the clinicaltrial.gov database since 2008. Among these trials, 59% target the extracellular domain of IGF-1R by mAb (Table 1); preliminary results from many of these phase 3 trials have been negative. Full reports of some of these trials have not yet been published. Trials using TKIs in breast cancer have also been reported. Most of the developed drugs have been competitive inhibitors of the ATP-binding site in the intracellular domain of the receptor. Compared with the mAb, these drugs are not selective for IGF-1R, but have roughly equipotent activity against IR.^67^ In clinical trials, single-agent activity of lisitinib (OSI-906) was reported,^68, 69^ but development was discontinued (Table 2). BMS-754807 development was discontinued in phase 2 trials without reporting results.^70^

**Table 2:** Toxicities associated with anti-IGF-1R therapy

Compound type					
	Estimated enrolled patients	Metabolism and nutrition disorders (grade 3 and 4)	Hyperglycemia (grade 3 and 4)	Clinical trial evolution	Reference ID # Clinical Trials.gov
*IGF-IR mAbs*					
Figitumumab (CP-751,871)	115	47.91%	52.08.48%	Terminated at Phase II	NCT00372996 NCT00976508
Cixutumumab (A12)	19	43.75%	56.24%	Terminated at Phase II	NCT00684983
Dalotuzumab (MK0646)	11	(–)	(–)	Terminated at Phase II	NCT00903006
AVE1642	18	(–)	(–)	Terminated at Phase II	NCT00774878
*Non-ATP antagonist TKIs*					
Linsitnib (OSI-906)	11	70.58	29.41%	Terminated at Phase II	NCT01205685

*Note:* (–) Indicates there are insufficient data or no data have been reported

Another IGF-1R inhibitor without direct ATP-binding activity, AXL1717 (Picropodophyllin) or PPP, has shown potential therapeutic characteristics in non-small cell lung cancer patients.^71^ This agent showed activity in mouse models of breast cancer^72^ but was not tested in the breast cancer. Several other clinical trials targeting IGF-1R have been reported as failures in phase III studies; therefore, many sponsors have terminated their development.^37^ The rationale behind this pharmacological inefficiency may be due to the dual role of both IGF-1R and IR-A in mediating ligand responses. By not targeting IR-A, mitogenic and survival pathways activated by IGF ligands, particularly IGF-2, may persist. Despite these failures in phase III trials, many phase I or II reports described exceptional responses to anti-IGF-1R antibodies as single agents. It could be hypothesized these responding tumors lacked IR-A expression. Thus, understanding the results of these clinical trials requires a more comprehensive development of predictive biomarkers.

## Rationale for targeting IGF-1R in drug-resistant breast cancer

Several strategies in breast cancer treatment have attempted to overcome major resistance mechanisms that include multi-drug resistance (MDR),^73^ hormone therapy acquired resistance,^74^ and resistance to targeted drugs. MDR frequently invokes efflux mechanisms for small molecule inhibitors as well as enhancement of anti-apoptotic pathways. Hormone therapy acquired resistance occurs after the suppression of ER function with selective estrogen receptor modulators (SERMs) such as tamoxifen^75^ or by lowering serum estradiol (E2) levels or blocking peripheral conversion of adrenal precursors with aromatase inhibitors.^76^ The third mechanism is TKI acquired resistance such as in HER2-targeted therapy.^77^ The complexity of the molecular mechanisms underlying these important clinical phenotypes remains a principal challenge for the development of new drugs.^78^ Development of resistance promotes the acquisition of activation of other oncogenic molecules, which result in phenotypic changes such as inhibition of apoptosis signaling, alteration of cell differentiation, and cell cycle regulation.

This growing need to identify novel targets inhibiting resistance to anti-breast cancer agents has made IGF-1R a potential candidate.^79, 80^ Data have shown the upregulation of IGF-1R as an alternative pathway utilized by breast cancer cells to escape the consequences of chronic exposure to different therapeutics. This upregulation of IGF-1R as a favorable alternative pathway to escape resistance has been observed with TKI targeting epidermal growth factor receptor,^81^ HER2,^82^ chemotherapy,^83^ and hormonal therapy.^84^ Based on this phenomenon, trials were developed to combine anti-IGF-1R with a targeted appropriate treatment as follows: anti-IGF-1R plus chemotherapy for TNBC, anti-IGF-1R plus hormonal therapy such SERMs for ER or PgR positive, and anti-IGF-1R plus aromatase inhibitor for postmenopausal patients. Many of these preclinical concepts have been tested in clinical trials in breast cancer, most studies have focused on endocrine sensitive and resistant breast cancers.

## Therapeutic strategies for IGF-1R network

Advanced studies in cell biology and biochemistry have characterized IGF-1R with IR and their isoforms and hybrids receptors operate as a complex network in the cytoplasmic membrane (Fig. 1). As previously described in this review, IGF-1 and IGF-2 interactions with their receptors are modulated by IGFBPs.^85^ Although the major signaling pathways activated by the IGF-1R have been identified, no drugs have yet been successful in clinical trials to target IGF-1R network in breast cancer therapeutically. While TKIs are promising agents, their disruption of glucose homeostasis and other metabolic functions make them difficult to employ as long-term clinical strategies.^70^ There are three principle challenges associated with anti-IGF-1R hormonal therapy: (1) most of the anti-IGF-1R antibodies are reported to have minor responses,^86^ (2) the therapy targeting IGF-1R causes hyperglycemia due either to disruption of GH homeostasis or direct inhibition of IR by TKIs,^87^ and (3) chronic exposure to anti-IGF-1R might induce resistance.

We have previously demonstrated exposure of MCF-7 breast cancer to an IGF-1R-ATP antagonist inhibitor. NVP-AEW541 induced resistance.^88^ These resistant breast cancer cells have shifted their dependency from IGF-1R to an alternative receptor tyrosine kinase (RTK) Tyro-3 to regulate cell growth and survival.^89^ Further analysis indicated mTOR/p70S6K controlled the expression of Tyro-3 independently of AKT. To enhance the effects of inhibiting IGF-1R, investigators have proposed the addition of chemotherapy to improve the potency of targeting IGF-1R.^90^ The combination of hormonal therapy plus an anti-IGF-1R moAb, e.g., ganitumab in metastatic breast cancer was one of the promising therapeutic strategies; unfortunately, hyperglycemia and hyperinsulinemia were major obstacles for the development of this drug.^33^ As discussed, IR is an essential component of the IGF-1R network, and IGF-1R mAb do not completely block its activation.

To avoid hyperglycemia and hyperinsulinemia due to IGF-1R antibodies, the I-SPY 2 trial has used ganitumab in combination with the antihyperglycemic agent metformin (NCT01042379). In several clinical trials combining anti-IGF-1R and endocrine treatment^33^ or chemotherapy with IGF-1R mAb figitumumab^90^ have failed to improve clinical outcomes, perhaps due to upregulation of insulin. Other trials have supported the strategy of blocking mTOR/p70S6K as a method to control insulin action and potentially avoid upregulation of IGF-1R signaling by inhibition of mTOR.^91^ While preclinical data support the concept that mTOR inhibition can overcome insulin stimulation of breast cancer,^92^ the combination of anti-IGF-1R with mTOR inhibitors (Table 1) to suppress further signaling^93^ has proven to be too toxic to pursue.

It has been shown that IGF-1R activation in breast cancer results in the tyrosine phosphorylation of IRS-1/2 and Src-homology-2-domains.^94^ These molecules are believed to be part of multiple additional tumorigenic adapter proteins and molecules such as Shc, Gab, Crk, and PI3K,^95^ which are recruited by IGF-1R network receptors. Among the therapeutic strategies aimed to target individual molecules associated with IGF-1R network, PI3K inhibitors are classified as valuable candidates.^96^ Clinical trials evaluating combinations of PI3K inhibitors and hormonal therapy (NCT01296555, NCT01870505) or other anti-cancer agents (NCT02051751, NCT01822613) are underway against breast cancer. These combinations might prevent the feedback loop^97^ and cross talk with RTK due to the single inhibition of PI3K.

Since IRS-1 is required for IGF-1R stimulation of cell proliferation and IRS-2 is involved in cancer motility and metastasis,^98^ inhibition of their function or expression could be therapeutically exploited. In addition to PI3K, IRS-1/2 has become another potential target against the IGF-1R network in breast cancer. Studies have demonstrated the dissociation of IRS-1/2 from IGF-1R by tyrphostin NT (NT152, NT75, NT157, NT205) inhibitors. Particularly NT157^99^ led to the irreversible IRS-1/2 protein elimination and cell growth inhibition in melanoma cells.^98^ Additional studies have confirmed the pharmacological effect of NT157 in osteosarcoma^100^ and prostate cancer.^101^ Although the therapeutic activity of NT157 against breast cancer has been discussed in several meetings, no clinical data have yet been published.

## Conclusion

In this review, we have described the role of IGF-1R and IR and their ligands, insulin, IGF-1, and IGF-2, to regulate cell growth, survival, and glucose uptake^102^ in breast cancer. The receptor system is complex, IR and IGF-1R genes can form several types of hybrid receptors such IGF-1R/IR-A, IGF-1R/IR-B, IR-A/IR-B, and others.^37^ All these IGFs-associated receptors must be considered when targeting the IGF-1R network. Although the partial amino acid sequences similarities are key factors of the functional resemblance of these receptors, the molecular interaction between these receptor families ultimately determines cellular effects of ligand activation as their affinities for the three ligands differ.

Since the IGFBPs’ family ensures the bioavailability of IGF-1/2 and insulin in serum, IGFBPs serve as another regulator of the IGF-1R network. Therefore, the variation in IGFBPs expression is a crucial biomarker in breast tissue, for instance, high IGFBP-3 in TNBC is associated with poor prognosis,^103^ while the loss of IGFBP-3, in vivo models, was associated with tumorigenic transformation.^104^ These interactions between ligand and IGFBPs have not been evaluated as either a predictive factor for IGF-1R-targeted therapies or as a potential therapeutic strategy to neutralize IGF action. Indeed, IGF-1R is the most targted molecule in IGFs pathways that was tested with several different approaches in cancer.^105^ Although mAb against IGF-1R have shown single-agent activity, their combination with other therapies has not been promising (Table 1). All of these trials were done in the absence of selective markers; thus, there is still a need for the incorporation of predictive biomarkers in the design of anti-IGF-1R network clinical trials. Additionally, there may be other ways to target the network.

PI3K is one of the major molecules interacting with IGF-1R to regulate cell signaling; the mutation of PIK3CA can lead to tumorigenesis in the absence of the suppressor PTEN.^106^ This makes PI3K a potential target, but the pharmacodynamics of PI3K inhibitors in breast cancer patient is not yet known. Our published data suggest another IGF-1R-associated molecule, IRS protein, believed to interact with PI3K, is critical in determining response to receptor activation.^16^ Thus, targeting of IRS proteins for degradation with compounds such as NT157 may provide a means to interrupt IGF and insulin signaling.

As IRS-1 and IRS-2 are thought to mediate most of the effects of IR and IGF-1R in breast cancer cells, it may be possible to disrupt this molecule without affecting normal glucose homeostasis mediated by other adapter proteins in insulin target organs. Ongoing investigation in our laboratory has motivated us to hypothesize that the molecular composition of plasma membrane microdomains associated with IGF-1R network receptors might dictate the tumorigenic activity of IRS-1/2 in breast cancer. Therefore, a comprehensive analysis of these complexes including their interaction with adapter proteins and serum ligands should result in the optimization of anti-IGF strategies in breast cancer.
